# Production, optimization, and purification of serratiopeptidase from *Bacillus siamensis* found in marine sediments

**DOI:** 10.3389/fmicb.2026.1817064

**Published:** 2026-04-23

**Authors:** Aswin Viswan, Anice Jossia Hermon, Anjana Hemabindu Madhusoodanan, Sreelakshmi Ramu Nair, Sinsha Kanisankandy, Kothari Neeti Suresh, Mohanasrinivasan Vaithilingam, Subathra Devi Chandrasekaran

**Affiliations:** School of Bio Sciences and Technology, Vellore Institute of Technology, Vellore, India

**Keywords:** anti-inflammatory, *Bacillus siamensis*, optimization, purification, Serratiopeptidase

## Abstract

Serratiopeptidase is a proteolytic enzyme with significant clinical applications, specifically acting as a potent anti-inflammatory agent. The current study focuses on the optimization of different process parameters for the enhanced production of serratiopeptidase from marine *Bacillus siamensis*. The effect of pH, metal ions, surfactants, and inhibitors on enzyme activity was also analyzed. The enzyme was purified by ammonium sulfate precipitation, dialysis, and gel filtration chromatography. Growth kinetics of the bacterial strain showed a maximum of 3.449 U/mL of enzyme production at 18 h. The maximum growth rate and enzyme activity were obtained when the optimized medium was supplemented with lactose as the carbon source (1.779 U/mL) and yeast extract as the nitrogen source (1.807 U/mL), at a pH of 7.2 (1.589 U/mL) and an inoculum size of 250 μL (2.5 × 10^7^ CFU/mL; 2.87 U/mL), respectively. The enzyme activity was highly stable at pH 6 (0.32 U/mL), in the presence of 20 mM CaCl₂ (0.45 U/mL), and 20 mM MgSO_4_ (5.5 U/mL). After purification of the protein, a 3.6-fold increase in the specific activity of the enzyme was attained. Sodium dodecyl sulfate–polyacrylamide gel electrophoresis (SDS–PAGE) analysis showed a band of molecular size of 50 kDa, confirming the presence of the anti-inflammatory protein. Fourier transform infrared spectroscopy (FTIR) analysis revealed the molecular and structural integrity of the protein. Serratiopeptidase produced from marine *Bacillus siamensis* was found to be highly stable and effective. The findings of this study support the potential of this anti-inflammatory protein as a potent candidate for therapeutic and industrial applications.

## 1 Introduction

Serratiopeptidase is a well-known proteolytic enzyme that has gained considerable attention in the industrial and pharmaceutical sectors. It is a zinc-containing metalloprotease enzyme with a molecular weight ranging from 45 to 60 kDa, known to be produced by *Serratia marcescens*, a Gram-negative opportunistic pathogen (Jadhav et al., 2020). It is used to treat several health conditions, such as inflammation, carpal tunnel syndrome, and wound healing (Bhagat et al., 2013). It is also used in the treatment of diseases, such as atherosclerosis, uterine fibroids, Alzheimer’s disease, and sinusitis. The main properties of serratiopeptidase include analgesic, anti-inflammatory, and anti-edema effects. Additionally, because of its strong thrombin-dissolving properties, it is used to treat and prevent thrombosis (Meng et al., 2023).

Since serrapeptase has extensive applications in treating inflammation, the demand for this enzyme is high. Several organisms, such as *Serratia liquefaciens*, *Bacillus licheniformis*, and *Bacillus subtilis*, have also been reported to produce serrapeptase (Bhagat et al., 2013). However, many of these organisms are pathogenic; hence, several studies have focused on the production of recombinant serrapeptase by cloning it in *Escherichia coli*, thereby reducing the risk of infection (Srivastava et al., 2019).

*Bacillus siamensis*, isolated from marine sediments in Ramanathapuram and Mandapam, Tamil Nadu, was found to be a potential serrapeptase-producing organism. Being non-pathogenic and derived from marine sediments, this organism has potential applications (Srivastava et al., 2019). It can be optimized for enhanced production of the serratiopeptidase enzyme and increased yield, enabling its use for large-scale commercialization of industrial requirements.

Different methods, such as ammonium sulfate precipitation, membrane dialysis, chromatography, and analytical techniques like FTIR (Fourier transform infrared spectroscopy) and HPLC (high-performance liquid chromatography), can be employed to assess purification fold and enzyme characterization, respectively. Additionally, stability studies focusing on the effect of pH, temperature, and ionic concentration can help in understanding enzyme performance under different conditions, which is very helpful for formulation as well as other industrial and pharmaceutical applications.

Studies have been conducted to enhance the volumetric productivity of serratiopeptidase from *Serratia* strains. Kumar and Bhattacharya (2025) optimized *Serratia marcescens* SP6 to achieve 2,495 U/mL of enzyme production, indicating a 10.16-fold improvement over basal serratiopeptidase activity, using 10 g/L casein and 18.4 g/L dextrose at pH 7. A study conducted by Nair and Devi (2025) in Serratia marcescens VS56, under optimized conditions of 3 g/L glucose, 3 g/L beef extract, and pH 7.5, yielded 6,516.4 U/mL serratiopeptidase, showing a 63.65% improvement over 3,981.87 U/mL obtained under basal conditions. Nair and Subathra Devi (2024) optimized *Serratia* sp. soil isolate VS56 to achieve 591 U/mL serratiopeptidase production in 26 h at 37 °C and pH 7, showing a 5.5-fold improvement over other microbial strains.

While these studies demonstrate the progress in enzyme production, the pathogenicity of *Serratia* necessitates the exploration of alternative sources. The present study aims to produce serratiopeptidase from a strain isolated from marine sediments. Previous studies have shown that *Serratia* spp. are the most dominant strain used in commercial enzyme production, whereas relatively few studies have reported on *Bacillus* spp. Therefore, this study presents the enhanced production of serratiopeptidase from *Bacillus siamensis* isolated from marine sediments.

## 2 Materials and methods

### 2.1 Bacterial strain

The enzyme was extracted from *Bacillus siamensis* VITAAA3, isolated from marine sediments (GenBank Accession Number PP077310) (Viswan et al., 2025).

### 2.2 Growth kinetics of *Bacillus siamensis*

To determine the growth phase of the bacteria and the peak time of enzyme activity, growth kinetics was performed as per the protocol described by Vinayagam et al. (2015) and Iyer et al. (2024). Tryptic soy broth was prepared in a side-arm flask and sterilized. A total of 100 μL (1 × 10^7^ CFU/mL) of seed culture was inoculated, and 2 mL aliquots were collected at every 2 h intervals for 30 h. Growth density was recorded at 600 nm, and a growth curve was determined. Enzyme activities, including anti-inflammatory activity and casein hydrolysis assays, were carried out according to the protocols described by Joseph et al. (2013) and Cupp-Enyard (2008), respectively, for each aliquot collected at 2 h intervals.

### 2.3 One-factor optimization

For the enhanced production of serratiopeptidase, one-factor medium optimization was carried out according to a modified protocol described by Wagdarikar et al. (2015). An azo casein assay, using azo casein as the substrate, was used to estimate enzyme activity. All assays were performed in triplicate, and standard deviation (SD), error values, analysis of variance (ANOVA), and t-tests were calculated.

#### 2.3.1 Optimization of carbon source

Different carbon sources, including dextrose, sucrose, glycerin, and lactose, were selected as alternative carbon sources for the tryptic soy broth (TSB) medium. A total of 0.25 g of each carbon source was added to 100 mL of medium. The medium was inoculated with a seed culture of 10^6^–10^7^ CFU/mL and incubated at 37 °C for 28 h.

#### 2.3.2 Optimization of nitrogen sources

For optimizing the nitrogen sources, yeast extract, tryptose, peptone, and ammonium sulfate were selected. Approximately 0.5 g of each nitrogen source was added to the medium as a substitute for soya peptone, which is one of the nitrogen sources in TSB medium. The medium was inoculated with 10^6^–10^7^ CFU/mL of seed culture and incubated at 37 °C for 28 h.

#### 2.3.3 Optimization of pH

For optimizing the pH value for the medium, different pH values, namely pH 4, pH 7, pH 10, and pH 14, were selected as alternatives to pH 7.2, which is the pH of the TSB medium. The medium was inoculated with 10^6^–10^7^ CFU/mL of seed culture for each pH range and incubated at 37 °C for 28 h.

#### 2.3.4 Optimization of inoculum size

Inoculum sizes such as 50 μL (5 × 10^6^ CFU/mL), 100 μL (1 × 10^7^ CFU/mL), 150 μL (1.5 × 10^7^ CFU/mL), 200 μL (2 × 10^7^ CFU/mL), and 250 μL (2.5 × 10^7^ CFU/mL) were selected. Each TSB medium was inoculated with the selected volume of seed culture and incubated at 37 °C for 28 h. Tryptic soy broth (composition including casein peptone, soya peptone, NaCl (sodium chloride), dipotassium hydrogen phosphate, and glucose) served as the control. After incubation, the growth density was recorded at 600 nm, and the supernatant was collected after centrifugation at 5,000 rpm for 20 min. Enzyme activities, including anti-inflammatory activity and casein hydrolysis assays, were carried out to determine the suitable carbon and nitrogen sources, as well as the optimal pH and inoculum size.

### 2.4 Partial purification of serratiopeptidase

For partial purification, ammonium sulfate precipitation was carried out at 20–70% saturation. A freshly prepared culture supernatant was used. Ammonium sulfate was added to the supernatant with constant stirring. After precipitation, the contents were centrifuged at 5,000 rpm for 20 min, and the resultant pellet was dissolved in phosphate buffer (pH 7) and dialyzed using a dialysis membrane (50) in the same buffer for 4 h, followed by buffer replacement. The setup was kept for overnight dialysis. After this procedure, the enzyme was transferred into a sodium borate–hydrochloric acid buffer (pH 9) and dialyzed at 4 °C overnight. The resultant dialysate was regarded as the partially purified enzyme (Devi et al., 2013; Nageswara et al., 2019; Archana et al., 2018).

### 2.5 Purification of the serratiopeptidase enzyme

Gel filtration chromatography was used for the purification of the enzyme. The column was packed with Sephadex G-50 (40 × 2 cm). The column was pre-eluted with phosphate buffer (pH 7), and then 2 mL of the partially purified enzyme was loaded onto the column and allowed to elute to separate protein fractions (Natarajan and Subashkumar, 2023). Thirty fractions of 0.5 mL each were collected in Eppendorf tubes, and the presence of protein in each fraction was confirmed using Lowry’s method of protein estimation with bovine serum albumin as the standard (Lowry et al., 1951). The Oda–Murao method for protease activity was also performed, with tyrosine as the standard, following a modified protocol described by Oda and Murao (1974). The purified enzyme was then characterized by SDS–PAGE and FTIR according to the protocols described by Simpson (2006) and Haris and Severcan (1999), respectively.

### 2.6 One-step purification of serratiopeptidase by the three-phase partitioning (TPP) method

The TPP method was used for the one-step purification of serratiopeptidase. Fresh seed culture (3 × 10^6^ CFU/mL) was inoculated into tryptic soy broth and incubated for 28 h. The culture was then centrifuged at 5,000 rpm for 20 min, and ammonium sulfate (30%) and an equal volume of t-butanol were added to the supernatant. The contents were then kept in a shaking incubator for 28 h at 37 °C (Babagil and Nadaroglu, 2022).

### 2.7 Stability study of serratiopeptidase

Factors affecting enzyme stability include pH, temperature, surfactants, and ionic concentration. Enzyme stability is an important parameter, and it is important to determine stability under different conditions. Different factors were selected to evaluate enzyme stability (Wagdarikar et al., 2015).

#### 2.7.1 Effect of pH

Different buffer systems were prepared, including 50 mM citrate phosphate buffer (pH 4), 50 mM phosphate buffer (pH 6), and 50 mM Tris–HCl (pH 8). To each buffer, 100 μL of purified enzyme was added and incubated for 30 min at 37 °C, followed by determination of protease activity using azo casein as the substrate (Oda and Murao, 1974).

#### 2.7.2 Effect of calcium chloride

Different concentrations of calcium chloride were prepared (1, 3, 10, 15, and 20 mM). To 1 mL of each concentration, 100 μL of purified enzyme was added and incubated at 37 °C for 30 min. The samples were then subjected to a protease assay.

#### 2.7.3 Effect of metal ions

Solutions of 20 mM of MgSO_4_, ZnSO_4_, FeCl_3_, MnSO_4_, and BaCl_2_ were prepared. To 1 mL of each solution, 100 μL of purified enzyme was added and incubated at 37 °C for 30 min. Thereafter, a protease assay was performed.

#### 2.7.4 Effect of surfactants and inhibitors

Surfactants (0.1%), including Tween-20 and Triton X-100, as well as 1 mM SDS, were prepared. To 1 mL of each solution, 100 μL of purified enzyme was added and incubated at 37 °C for 30 min. Subsequently, a protease assay was carried out. To evaluate the effect of inhibitors, 0.1% dimethyl sulfoxide (DMSO), ethyl methyl sulfonate (EMS), and 20 mM EDTA were prepared. To 1 mL of solutions,100 μL of purified enzyme was added and incubated at 37 °C for 30 min, followed by the protease assay.

#### 2.7.5 Statistical analysis

The ANOVA results revealed a highly significant difference among the group means (*F* = 2,398, *p* < 0.0001). Since the *p*-value is less than 0.05, the differences are statistically significant. All experiments were conducted in triplicate, and graphs were plotted using OriginPro 2023 and GraphPad Prism 10. Additionally, the model exhibited an excellent fit, with an R^2^ value of 0.9991.

## 3 Results

### 3.1 Growth kinetics of *Bacillus siamensis*

**Figure 1:**
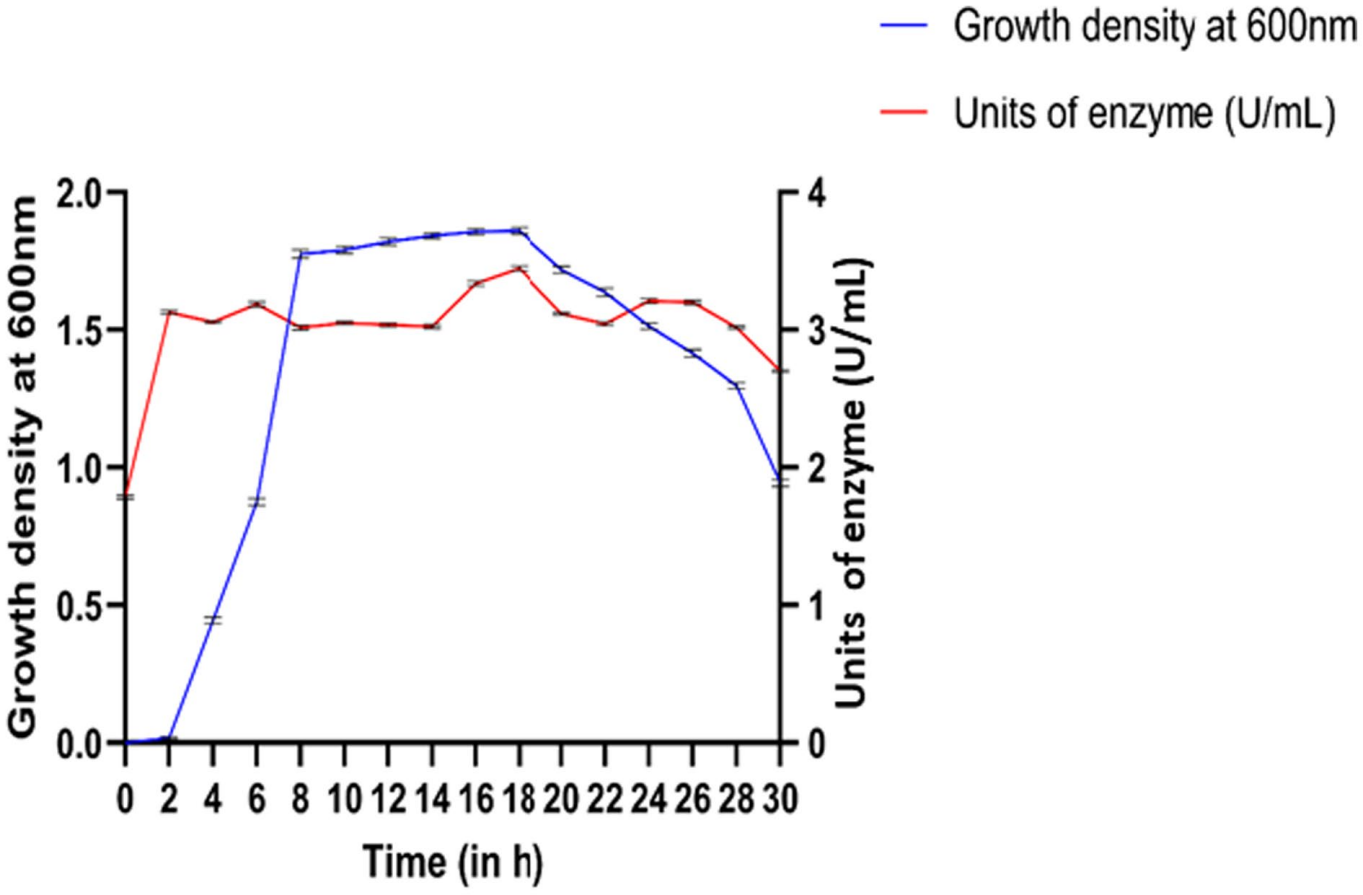
Growth curve of *Bacillus siamensis* with growth density and enzyme activity at different time intervals.

In the growth kinetics of *Bacillus siamensis*, growth increased from 2 h and remained in the log phase until 8 h, after which the organism entered the stationary phase until 18 h, followed by the decline phase (Figure 1). In the casein hydrolysis assay conducted at 18 h, enzyme activity was recorded to be 3.449 U/mL, which was higher compared to that at other time points. This suggests that protease activity was highest at this time point, leading to the breakdown of casein (Figure 1). The percentage protection of the enzyme is depicted, which shows that the anti-inflammatory activity of the enzyme is more predominant at 22 h of the growth period, corresponding to the stationary phase (Figure 1).

### 3.2 One-factor optimization

#### 3.2.1 Optimization of carbon source

**Figure 2:**
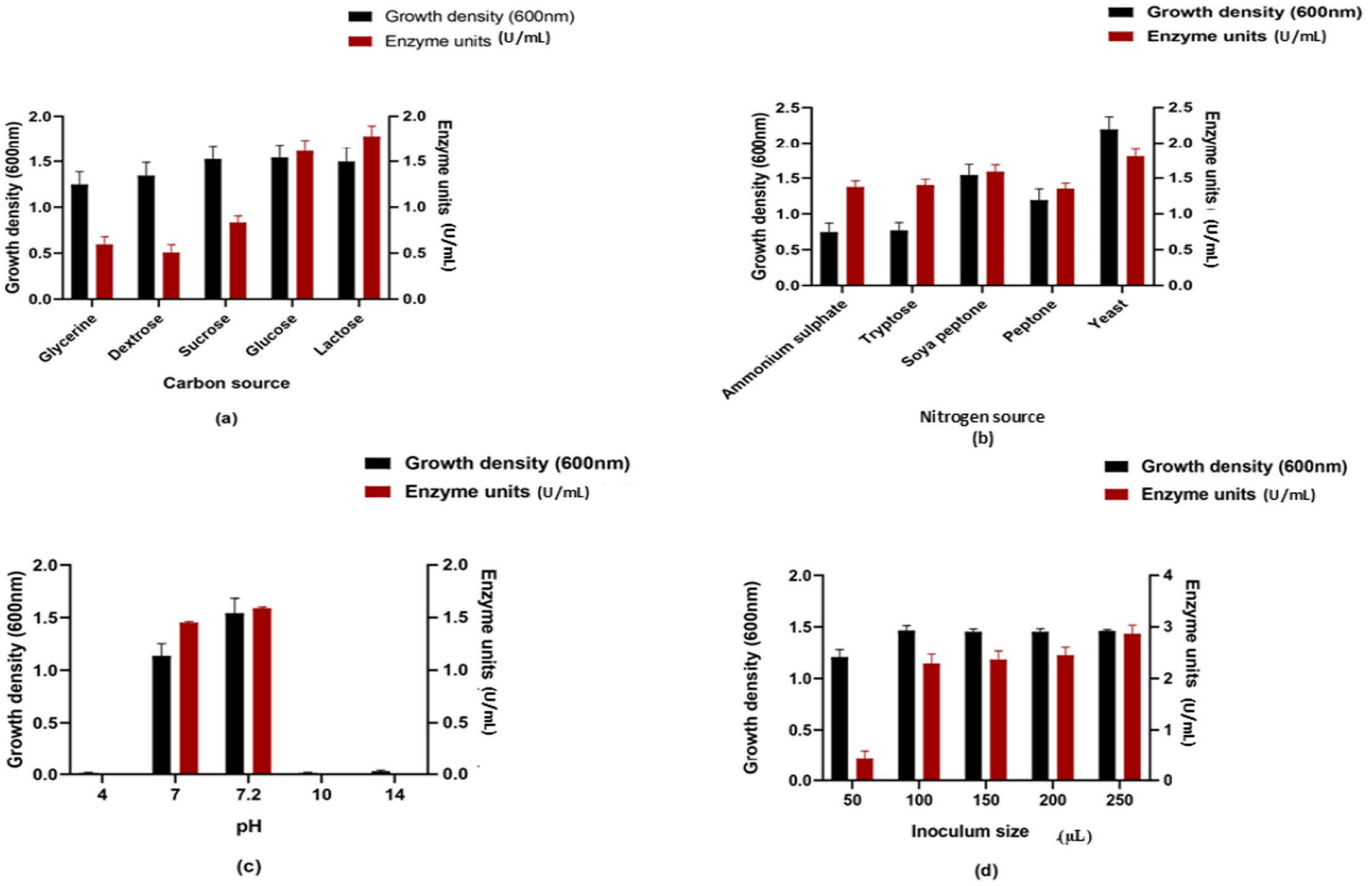
Enzyme activity and growth density at different **(a)** carbon source, **(b)** nitrogen source, **(c)** pH, and **(d)** inoculum size (μL).

**Figure 3:**
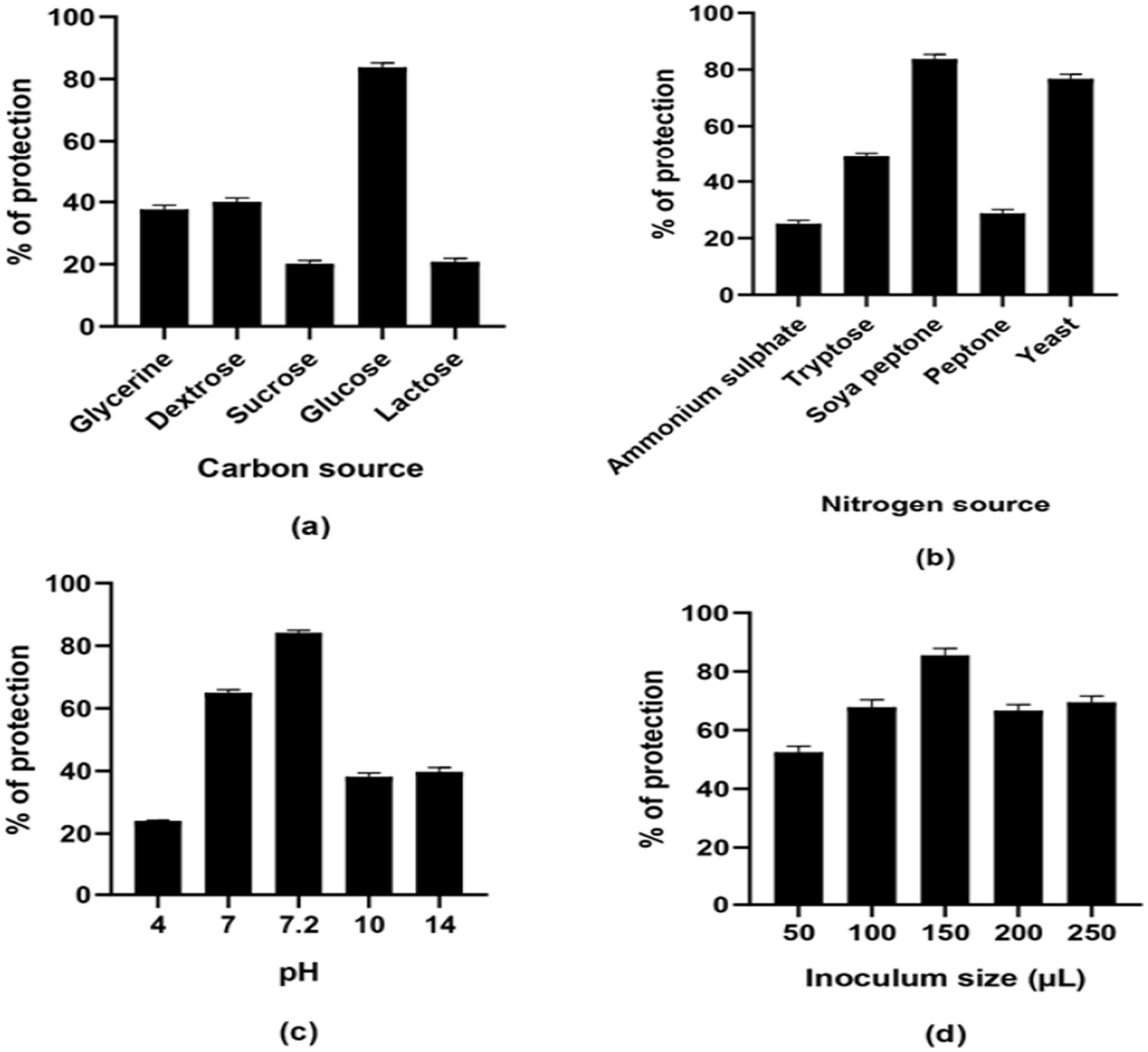
Anti-inflammatory activity of different **(a)** carbon source, **(b)** nitrogen source, **(c)** pH, and **(d)** inoculum size (μL).

The enzyme units present in the medium with lactose as the carbon source, after the casein hydrolysis assay, were higher than those observed with other carbon sources. When lactose was used as the carbon source, enzyme production was 1.779 U/mL. In the case of the second-best carbon source, glucose, enzyme activity was 1.589 U/mL. For sucrose, glycerin, and dextrose, enzyme activities were 0.835 U/mL, 0.595 U/mL, and 0.507 U/mL, respectively (Figure 2a). In the anti-inflammatory assay, glucose showed significant activity (83.75 ± 1.221%), which is a component of TSB (Figure 3a).

#### 3.2.2 Optimization of nitrogen source

The medium containing yeast extract as the nitrogen source exhibited higher enzyme units in the casein hydrolysis assay compared to media containing alternative nitrogen sources. In medium with yeast extract, *Bacillus siamensis* produced 1.807 U/mL units of enzyme. The second-best nitrogen source was soya peptone, which produced 1.589 U/mL. The lowest enzyme activity (1.354 U/mL) was observed with peptone. For tryptose and ammonium sulfate, enzyme activities were 1.402 U/mL and 1.377 U/mL, respectively (Figure 2b). Soya peptone, which is the nitrogen source of TSB, showed significant anti-inflammatory activity (83.75 ± 1.273%) compared to other nitrogen sources, whereas the medium containing yeast extract showed comparable activity (76.8 ± 1.266%) (Figure 3b).

#### 3.2.3 Optimization of pH

The casein hydrolysis assay and anti-inflammatory assay performed at different pH values showed significant results in the control (TSB) at pH 7.2, where the enzyme unit was highest compared to other pH values. Notable activity was also observed at pH 7. The enzyme activity was 1.589 U/mL and 1.452 U/mL at pH 7.2 and 7, respectively (Figure 2c). In the anti-inflammatory assay, the percentage protection was 84.2 ± 0.458% and 65 ± 0.568% at pH 7.2 and 7, respectively (Figure 3c).

#### 3.2.4 Optimization of inoculum size

The casein hydrolysis assay performed with various inoculum sizes showed the highest enzyme activity (2.87 U/mL) at a 250 μL concentration. The lowest enzyme activity (0.45 U/mL) was observed at 50 μL. For 100 μL, 150 μL, and 200 μL inoculum sizes, the enzyme activities were 2.289 U/mL, 2.37 U/mL, and 2.455 U/mL, respectively. In the anti-inflammatory assay, an inoculum size of 150 μL showed high activity (85.56 ± 0.125% protection). This suggests that higher inoculum size may enhance enzyme activity (Figure 2d).

These findings suggest that the optimum medium components for peak enzyme activity are lactose as the carbon source, yeast extract as the nitrogen source, 250 μL as the inoculum size, and pH 7.2 as the optimum pH value. These components were selected based on casein digestion activity rather than anti-inflammatory activity. The optimized medium components include lactose, yeast extract, NaCl, casein peptone, and dipotassium hydrogen phosphate at pH 7.2. The organism, when inoculated in the optimized medium at 37 °C, showed enhanced growth after 24 h. Growth density was also higher at 600 nm.

### 3.3 Purification of serratiopeptidase

The total protein content as well as the enzyme units were reduced after purification of the enzyme. The crude enzyme contained high protein content as well as enzyme units. The total protein content in the crude sample was 393 mg/300 mL. Following ammonium sulfate precipitation, dialysis, and gel filtration chromatography, the total protein was 189 mg/300 mL, 156 mg/300 mL, and 89.1 mg/300 mL, respectively. The specific activity increased after each purification step, and the purification fold also increased, indicating enrichment of serratiopeptidase (Table 1).

**Table 1:** Determination of specific activity and yield of the enzyme from *Bacillus siamensis*.

Fractions	Total protein (mg/300 mL)	Enzyme units (U/300 mL)	Total activity	Specific activity	Yield (%)	Purification fold
Supernatant	393.6	2,559	767,700	6.5	100	1
Ammonium sulfate precipitation	189.6	2,124	637,200	11.2	83	1.73
Dialysis	156.3	2,109	632,700	13.4	82.4	2.07
Gel filtration chromatography	89.1	2,085	625,500	23.4	81.4	3.6

In one-step purification of serratiopeptidase, after 24 h of incubation, a white matte-like layer was observed floating in the middle layer of the solution. The upper layer consisted of organic t-butanol, the middle layer contained the interfacial precipitate, and the lower layer was the aqueous phase. The matte layer formed in the middle layer represented the purified serratiopeptidase obtained by the TPP method.

### 3.4 SDS–page

**Figure 4:**
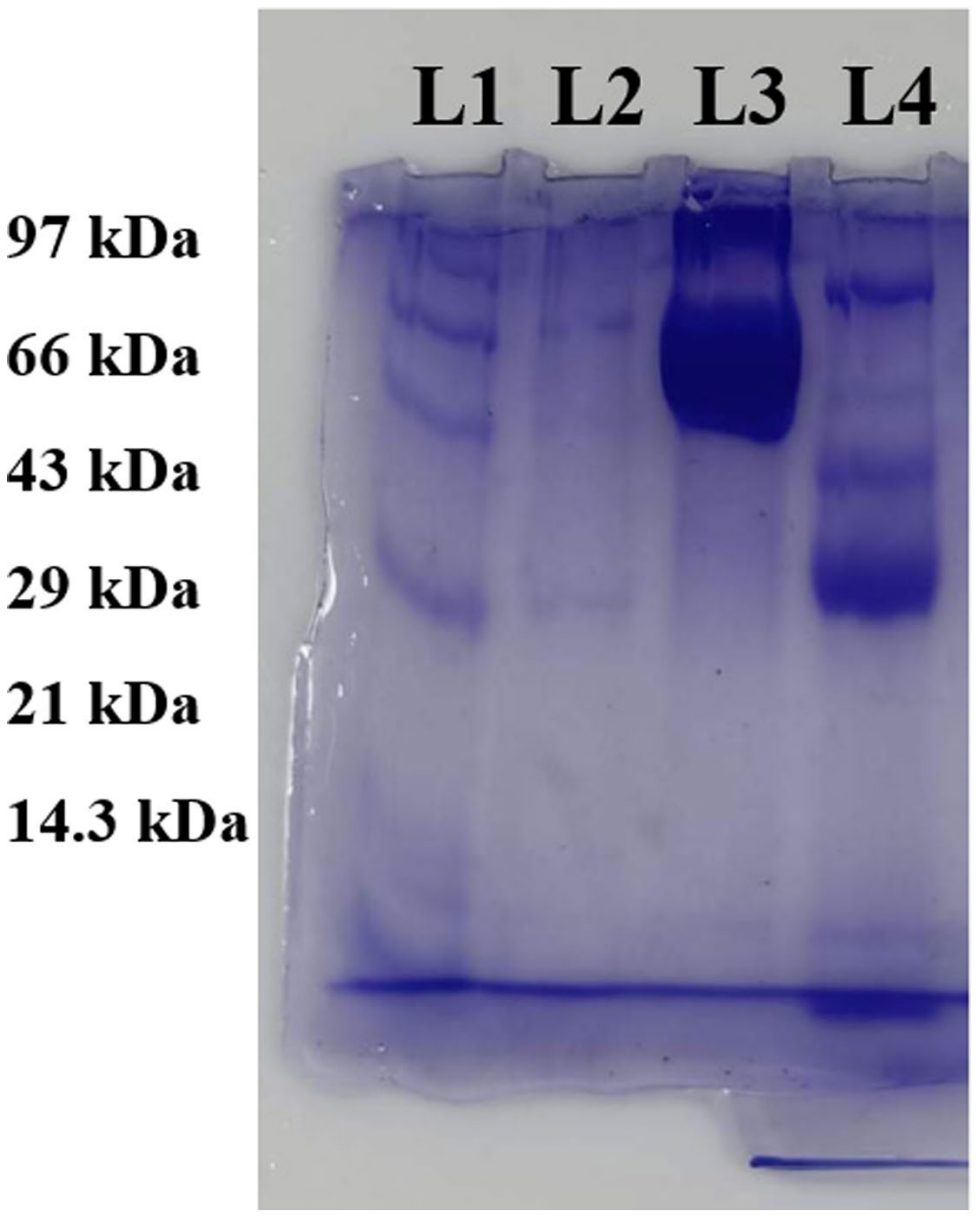
SDS–PAGE: lane L1: molecular weight ladder; lane L2: partially purified sample; lane L3: purified sample; and lane L4: crude sample.

The molecular weight determination of serratiopeptidase by SDS–PAGE revealed a band near 50 kDa, confirming the presence of the enzyme (Figure 4). The uncropped image is provided in Supplementary Figure S1.

### 3.5 FTIR analysis

**Figure 5:**
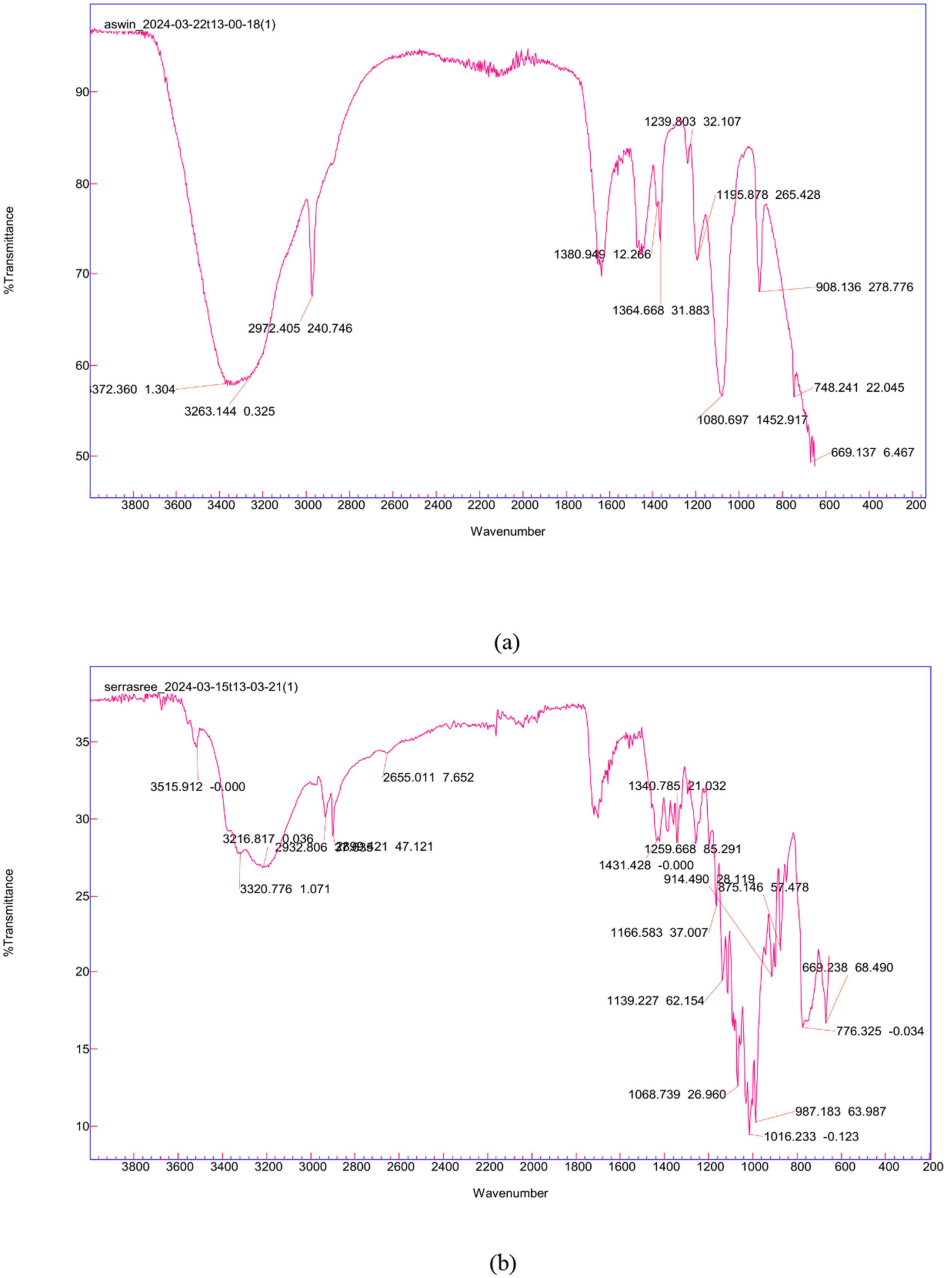
FTIR spectra of the protein samples **(a)** purified enzyme and **(b)** standard serratiopeptidase.

FTIR analysis of the sample showed similar functional groups in the signature region when compared with standard serratiopeptidase. Stretching was observed between 1,800–1,600 cm^−1^ and 1,600–1,400 cm^−1^ in the sample, corresponding to C=O, N=O, C=N, and C=C vibrations. Stretching between 1,200–1,000 cm^−1^ and 1,000–800 cm^−1^ indicated the presence of C–C, C–N, and C–O bonds, whereas stretching between 2,800–3,000 cm^−1^ and 3,000–3,400 cm^−1^ indicated C–H, O–H, and N–H vibrations. The similar functional group in the sample confirms the presence of the enzyme (Figure 5).

### 3.6 Stability study of serratiopeptidase

**Figure 6:**
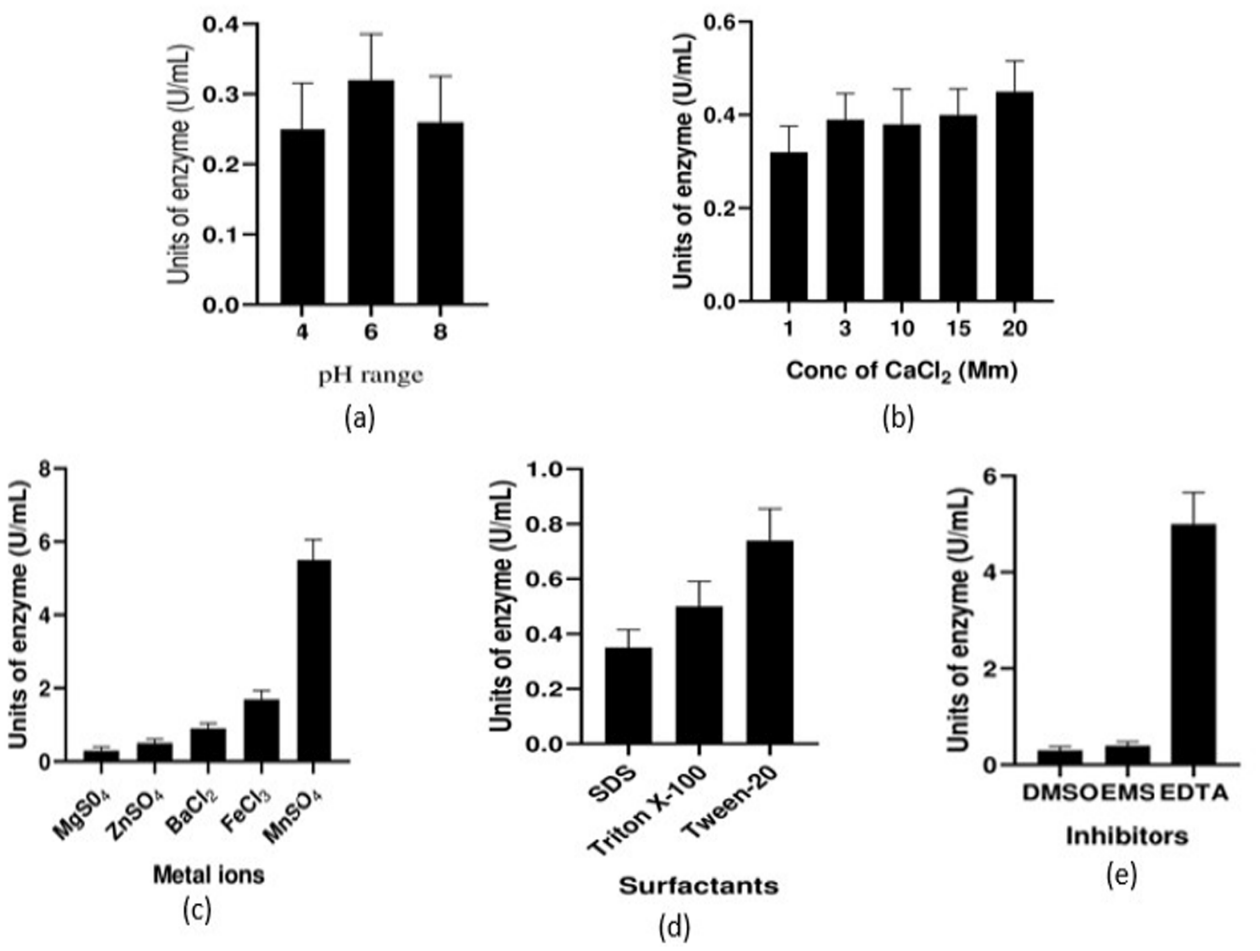
Stability of the enzyme at different **(a)** pH range, **(b)** concentration of CaCl_2_, **(c)** metal ion concentrations, **(d)** surfactant molecules, and **(e)** inhibitors.

The serratiopeptidase was found to be stable at pH 6 compared to pH 4 and 8, indicating its effectiveness in hydrolyzing azocasein at pH 6 (Figure 6a). Enzyme activity was highest at 20 mM calcium chloride; although comparable activity was also observed at 3 mM, 10 mM, and 15 mM CaCl_2_ (Figure 6b). This signifies that the enzyme is stable in these concentrations of CaCl_2_ solutions.

The stability study with different metal ions showed that enzyme activity was highest in the manganese sulfate solution. Notable activity was also observed with ferric chloride (Figure 6c). The experiment with surfactants revealed the efficiency of the enzyme against Tween-20, also, it showed some significant activity with Triton X-100 (Figure 6d). Among the inhibitors added, the enzyme activity was found in the presence of EDTA and not with EMS and DMSO (Figure 6e). The enzyme activities were 0.32 U/mL, 0.45 U/mL, 5.5 U/mL, 0.74 U/mL, and 5 U/mL at pH 6, 20 mM CaCl_2_, and in the presence of MnSO_4_ solution, Tween-20, and EDTA (Figure 6).

## 4 Discussion

*Bacillus* spp. are Gram-positive bacteria capable of producing a variety of enzymes. One among those enzymes is serratiopeptidase, which has anti-inflammatory properties. The different phases in the growth of *Bacillus siamensis* were observed to be lag, log, stationary, and decline phases, as in other bacteria (Madigan et al., 2008). There was exponential growth during the log phase, which lasted from 2 to 8 h. This showed that the bacteria were actively replicating. Favorable environmental conditions and nutrient availability are often associated with this phase as the optimal period for growth. After the log phase, the stationary phase continued until 18 h. At this stage, the number of bacterial cells growing and dying remains balanced. During this phase, resources become limited, and metabolic activity slows down as the population reaches a limit (Tortora et al., 2013). The biomass obtained was much higher at 10 and 18 h than at other times. This may indicate that the organism’s metabolism changed or that it adapted to environmental conditions. Based on this result, *Bacillus siamensis* may have changed its metabolism during these times, possibly to make better use of resources or respond to environmental cues (Madigan et al., 2008).

A casein hydrolysis test conducted after 18 h showed a high optical density, indicating increased enzyme activity, which resulted in the breakdown of casein into amino acids and smaller peptides. In this phase, when bacteria are stationary, they often have more metabolic capacity to find nutrients, which may explain the increased enzyme activity at this stage.

A study conducted in *Serratia marcescens* SP6 by a one-factor-at-a-time approach and Plackett–Burman design detected that mainly three parameters, namely casein as the nitrogen source, dextrose as the carbon source, and initial pH of the medium, substantially altered serratiopeptidase production (Kumar and Bhattacharya, 2025). In various studies, optimal conditions for increased protease production were investigated across different *Bacillus* species. Glucose emerged as the preferred carbon source among four *Bacillus* species tested for enzyme production, surpassing sucrose, fructose, maltose, starch, and cellulose (Boominadhan et al., 2009).

Notably, *Bacillus subtilis* exhibited increased activity in glucose-rich environments, while *Bacillus licheniformis* showed activity in starch substrates (Moustafa et al., 1988). Beef extract (organic) and ammonium carbonate (inorganic) were found to be the best options as nitrogen sources for protease production in certain *Bacillus* species (Shafee et al., 2005). These substrates facilitated high proteolytic activity after 48 h of incubation. Boominadhan et al. (2009) reported pH 8 as optimal for their test isolates. The studies on *Bacillus licheniformis* S-40 showed better results in the alkaline pH range of 7–12 (Shafee et al., 2005).

We mainly focused on the protease activity of the enzyme rather than the anti-inflammatory activity, and the medium components were also chosen based on protease activity. In a study conducted by Chander et al. (2021), it was found that during the partial purification process using ammonium sulfate precipitation at 30–80% saturation, a specific activity of 7,659 units/mg protein was observed (Chander et al., 2021). Subsequent purification through MonoQ ion-exchange chromatography yielded a higher specific activity of 20.492 units/mg protein. Even though MonoQ ion-exchange chromatography exhibited higher specific activity compared to ammonium sulfate precipitation, the total protein content was higher after ammonium sulfate precipitation (Chander et al., 2021).

Salarizadeh et al. (2014) suggested ammonium sulfate as an effective reagent for precipitation and concentration. They precipitated the metalloprotease produced by *S. marcescens* ZF03 using ammonium sulfate precipitation, which, after dissolving in a Tris buffer of pH 8, followed by dialysis, exhibited good purity. Different saturation levels of ammonium sulfate were used by them, among which 67% was the best. In Devi et al. (2013), the molecular weight determination of serratiopeptidase by SDS–PAGE confirmed that the molecular weight was 50 kDa. A similar study by Machielsen et al. (2006) also demonstrated the molecular weight of serratiopeptidase as 50 kDa (Devi et al., 2013). The acquired spectrum was compared to the documented FTIR spectra of serratiopeptidase, validating the purity of the produced enzyme as it exhibited identical peaks as described in a recent publication by Kaur and Singh (2015).

In a study conducted by Pakhale and Bhagwat (2016), a TPP assay was employed to assess the activity of serratiopeptidase. This assay involved the saturation of cell-free broth with ammonium sulfate and t-butanol, resulting in the formation of an interfacial precipitate layer between the upper t-butanol layer and the lower aqueous layer. Upon collection and subsequent mixing in 50 mM Tris–HCl buffer (pH 8.0), this precipitate layer demonstrated notable serratiopeptidase activity upon analysis (Pakhale and Bhagwat, 2016).

The formation of this interfacial precipitate layer serves as a crucial step in the TPP assay, as it enables the separation and concentration of the target enzyme from the complex mixture. In the examination conducted by Salarizadeh et al. (2014), the stability of metalloprotease sourced from *Serratia marcescens* sp. ZF03 was investigated. The experimentation included the utilization of inhibitors such as EDTA, PMSF, and iodoacetamide. It was observed that EDTA notably inhibited protease activity in contrast to the other inhibitors.

The influence of various metal ions on the metalloprotease activity was also explored. Specifically, Co^2+^ and Na^+^ ions were found to enhance metalloprotease activity. However, Fe^2+^, Fe^3+^, and Mn^2+^ ions, along with 2 mM PMSF, exhibited partial inhibition of protease activity. Optimal conditions for enzyme activity were found to be within the temperature range of 50–55 °C, with pH levels ranging from 8 to 10, yielding significant enzyme activity, peaking at pH 9 (Salarizadeh et al., 2014).

Metal ions, depending on their type and concentration, can influence enzyme activity. Surfactants affect enzyme stability by disrupting structure and function, as they are amphiphilic molecules. pH affects the ionization state of amino acid residues and influences enzyme conformation and activity. Calcium chloride can facilitate enzyme–substrate interactions or coordinate with certain amino acid residues to enhance enzyme stability. Different concentrations of calcium chloride can therefore affect the stability and activity of serratiopeptidase.

Recent research findings showed advancements in the optimization of serratiopeptidase production, with a greater focus on host organisms that are safe and suitable for industrial applications. Statistical tools such as response surface methodology and ANOVA have been shown to be effective in increasing enzyme production, as evidenced by recent research findings, where the production of the enzyme was maximized by optimizing the pH, temperature, and nutrient sources (Kumar and Bhattacharya, 2025; Nair and Devi, 2025). Similar to the study conducted by Luthra et al. (2025), where soybean flour and peptone were identified as effective carbon and nitrogen sources, respectively, the present study, with lactose and yeast extract as the respective nutrient sources, also shows the importance of nutrient selection for enhancing enzyme production.

Koul et al. (2024) have successfully shown the extracellular production and partial purification of serratiopeptidase from endophytic bacteria, supporting the possibility of discovering new microbial sources. Nair and Subathra Devi (2024) demonstrated the successful bioprospecting of a variety of environmental isolates with substantial proteolytic activity. This indicated the list of potential producers was different from *Serratia marcescens*. Attempts using non-pathogenic host organisms have gained attention, with *Bacillus licheniformis* showing a production level of 22.85 IU/mL under optimized media conditions (Wagdarikar et al., 2015), making it a potential host for the production of the enzyme, as opposed to the traditional *Serratia marcescens.*

The use of silkworm pupae as a substrate in fermentation by Melchor-Moncada et al. (2024) demonstrated a promising approach to enhance serratiopeptidase production from *Serratia marcescens* using Plackett–Burman design. In contrast, the present study used a one-factor-at-a-time approach, providing a simple, comprehensive optimization strategy, highlighting the importance of choosing the right optimization factors for fermentation processes. Overall, these studies have confirmed that even though native production is by *Serratia*, recent developments in strain diversification and bioprocess optimization are leading toward the establishment of efficient production platforms for serratiopeptidase.

## 5 Conclusion

Serratiopeptidase produced from *Bacillus siamensis* showed significant protease and anti-inflammatory activity. The present study mainly focused on the proteolytic activity of the enzyme rather than the anti-inflammatory activity. The results of the current study indicated that the nutritional composition and culture conditions have notable effects on enzyme activity. This study provides valuable insights for a better understanding of enzyme properties and stability, which could give detailed knowledge for its optimization for industrial and therapeutic applications. The marine strain *Bacillus siamensis* can be further exploited for its *in vitro* and *in vivo* anti-inflammatory activity and industrial production of the enzyme.

## Data Availability

The nucleotide sequence data of the organism used in the current study is deposited in the GenBank database under the accession number PP077310. https://www.ncbi.nlm.nih.gov/nuccore/pp077310.
