# Supplementary material for: Production, optimization, and purification of serratiopeptidase from Bacillus siamensis found in marine sediments
Source: Front Microbiol. 2026 Apr 23;17:1817064. doi: 10.3389/fmicb.2026.1817064 (PMC13149401; doi:10.3389/fmicb.2026.1817064)
Supplement: Supplementary file 1 [file Supplementary_file_1.docx]

Supplementary file


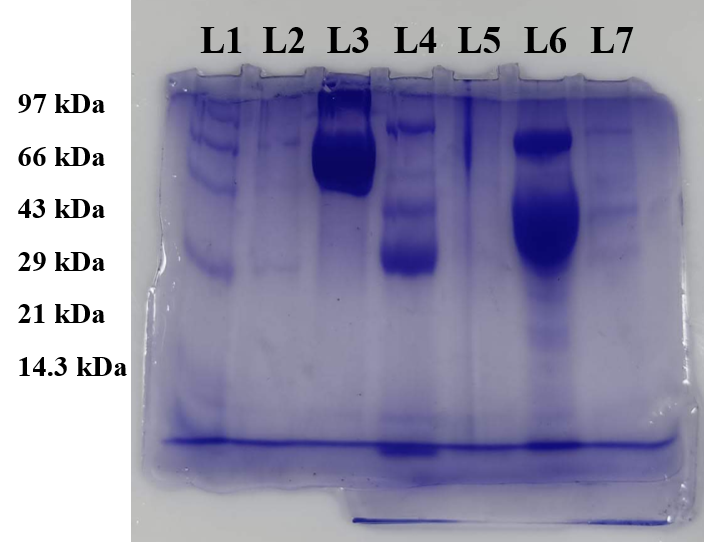


Fig: S1. Lane 1[L1] Ladder, Lane 2 [L2]- partially purified sample, Lane 3 [L3]- purified sample, Lane 4 [L4]- Crude sample, Lane 5 [L5], Lane 6 [L6] and Lane 7 [L7]

Only samples in L1, L2, L3 and L4 are part of this study. Samples in L5, L6 and L7 are not part of this study.
